# Function of the Active Site Lysine Autoacetylation in Tip60 Catalysis

**DOI:** 10.1371/journal.pone.0032886

**Published:** 2012-03-28

**Authors:** Chao Yang, Jiang Wu, Y. George Zheng

**Affiliations:** Department of Chemistry, Georgia State University, Atlanta, Georgia, United States of America; St. Georges University of London, United Kingdom

## Abstract

The 60-kDa HIV-Tat interactive protein (Tip60) is a key member of the MYST family of histone acetyltransferases (HATs) that plays critical roles in multiple cellular processes. We report here that Tip60 undergoes autoacetylation at several lysine residues, including a key lysine residue (i.e. Lys-327) in the active site of the MYST domain. The mutation of K327 to arginine led to loss of both the autoacetylation activity and the cognate HAT activity. Interestingly, deacetylated Tip60 still kept a substantial degree of HAT activity. We also investigated the effect of cysteine 369 and glutamate 403 in Tip60 autoacetylation in order to understand the molecular pathway of the autoacetylation at K327. Together, we conclude that the acetylation of K327 which is located in the active site of Tip60 regulates but is not obligatory for the catalytic activity of Tip60. Since acetylation at this key residue appears to be evolutionarily conserved amongst all MYST proteins, our findings provide an interesting insight into the regulatory mechanism of MYST activities.

## Introduction

Histone acetylation is an evolutionarily conserved post-translational modification mark on the chromatin template in eukaryotic systems. The reaction of acetylation is catalyzed by histone acetyltransferases (HATs) which fall into several major families based on sequence homology, including p300/CBP, PCAF/GCN5, Rtt109 and the MYST proteins [1], [2], [3], [4], [5]. On the chromatin template, histone lysine acetylation can loosen nucleosome structures and regulate chromatin-associated nuclear processes such as gene transcription and DNA repair [6], [7], [8], [9], [10], [11], [12], [13], [14], [15]. Furthermore, recent proteomics surveys showed that protein acetylation extends beyond the chromatin realm and regulates a variety of biological functions and processes, including cell cycle, cytoskeleton remodeling, chaperones, ribosome, and metabolic pathways [16], [17], [18], [19], [20], [21].

The MYST proteins are the largest family of HATs reported so far. They share a highly conserved MYST domain which is responsible for AcCoA binding and substrate acetylation. MYST HATs mediate diverse biological functions ranging from gene regulation, cell cycle regulation, dosage compensation [22], [23], genomic imprinting [24], to embryo development [25], [26]. Of note, many MYST proteins have been found to be deregulated in human diseases, especially leukemia, highlighting their pharmacological significance as attractive drug targets [2], [27], [28]. As a key MYST member, the 60-kDa Tat interactive protein (Tip60) was originally identified as a cofactor of HIV-1 Tat protein to regulate viral gene expression [29], [30]. Later on Tip60 was shown to be a MYST HAT and acetylate a wide array of protein targets including nucleosome core histones [31] and various transcriptional factors such as androgen receptor (AR) [32], ATM [33], [34], p53 [35], [36], Myc [37], STAT3 [38], NF-κB [39], [40], E2F [41], etc. Tip60 participates in the regulation of many important cellular processes [42], including apoptosis [43], [44], [45], DNA damage repair [9], [44], [46], [47], [48], developmental cell signaling [49], and ribosomal gene transcription [50]. Elucidation the mechanism of Tip60 activity regulation will provide a better understanding of its *in vivo* function in diverse signaling pathways. In this paper, we report that Tip60 is autoacetylated at a conserved lysine residue in the active site and the autoacetylation mediates the catalytic activity of the enzyme. Since this lysine is strictly conserved amongst all the MYST proteins, our findings reveal a novel mechanism of MYST activity regulation.

## Results

### Autoacetylation of the MYST HAT Tip60

**Figure 1:**
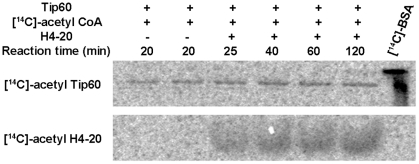
Phosphorimaging analysis of HAT reaction mixtures reveals autoacetylation of Tip60. Each reaction mixture contained 2.1 µM Tip60, 1.5 µM [^14^C]-AcCoA and 400 µM H4-20. Protein samples were resolved on the 20% SDS-PAGE. The gel was dried under vacuum and then exposed to the phosphorimage screen.

**Figure 2:**
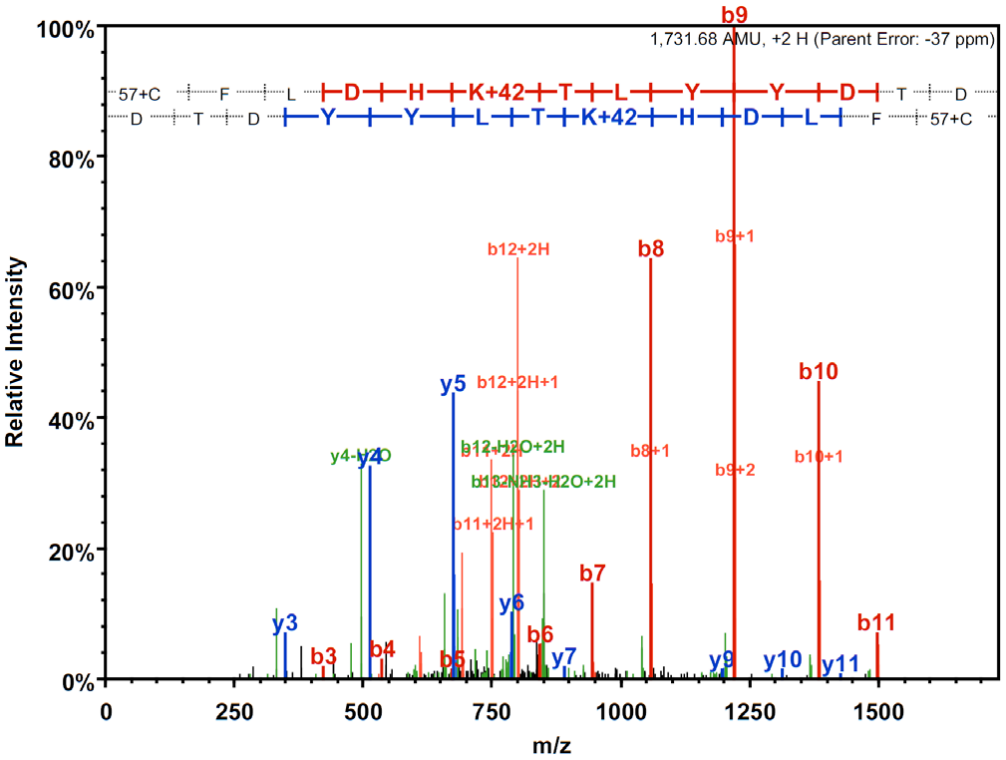
MS/MS analysis of the AA(320–334) sequence showing that Tip60 is acetylated at lysine 327.

**Figure 3:**
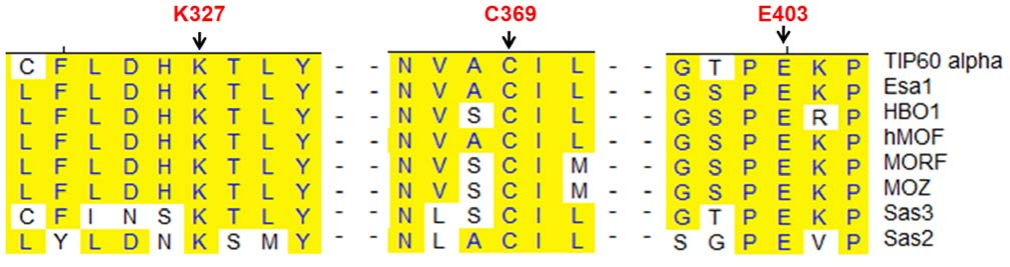
Sequence alignment showing that the three key residues are highly conserved in the active site of the MYST HATs. The numbering is based on Tip60 alpha.

Tip60 is an important MYST member that acetylates specific lysine residues in multiple protein substrates. To investigate the biochemical mechanism and function of Tip60-mediated acetylation, we expressed hexahistidine-tagged Tip60 in *E. coli* and purified the protein on Ni-NTA affinity beads. In one HAT assay experiment with the recombinant Tip60, we used a synthetic 20-aa histone H4 tail peptide (H4-20) as the substrate and [^14^C]-AcCoA as the acetyl donor. Following the HAT reaction, the reaction mixture was resolved on 20% SDS-PAGE and the radioactive gel was analyzed by phosphorimaging. As is clearly seen that on the autoradiograph, in addition to the acetylated H4 peptide band, Tip60 was also strongly acetylated (Fig. 1). Of note, the autoacetylation of Tip60 was also observed by other researchers [51], [52]. Key questions remain to be addressed as to where the autoacetylation sites are located, how the autoacetylation occurs, and how the autoacetylation of Tip60 is correlated to substrate acetylation. To clarify these important biochemical questions, we carried out a proteomic analysis to search for possible autoacetylation sites of Tip60. In the experiment, recombinant Tip60 protein was subjected to SDS-PAGE separation. The protein band was cut, cleaned, and dried under vacuum. The protein was then digested with AspN, LysC, and trypsin enzymes and each sample was analyzed by LC-MS/MS. From this study, we found that the Tip60 protein contained several acetylated lysine residues: K76, K80, K104, K150, K187, K327, and K383 (See Fig. 2 and Figures in Supporting Information S1). The most intriguing acetylated residue is K327 because this residue is located in the active site as revealed in the reported MYST HAT protein structures [53]. Importantly, this residue is strictly conserved among all the MYST HAT proteins (Fig. 3). A further evidence for this active site lysine autoacetylation is that in the recently reported X-ray Tip60 HAT domain structures, the same lysine residue was shown in the acetylated form (PDB 2OU2).

### The effect of K327 mutagenesis on the enzymatic activity of Tip60

**Figure 4:**
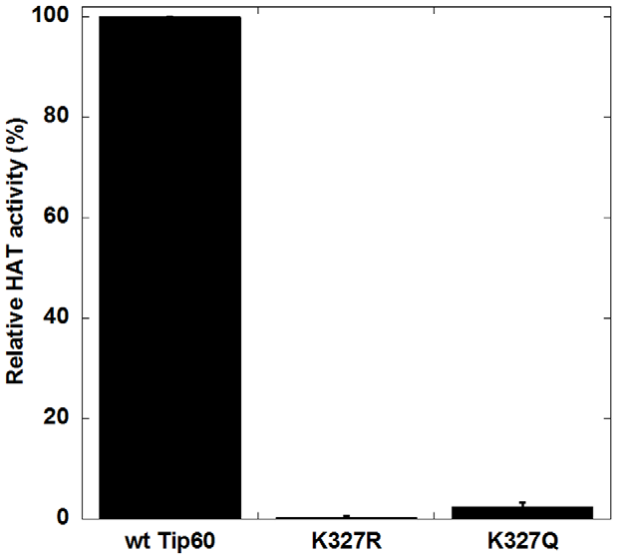
Effect of K327 mutation on HAT activity of Tip60. The HAT assays were performed at 30°C for 5 min and each reaction contained 10 µM [^14^C]-AcCoA, 200 µM H4-20, and 100 nM Tip60 protein.

The vicinity of the acetylated K327 (K327ac) to the active site suggests that this autoacetylation might directly regulate the enzymatic activity of Tip60. To understand the role of K327ac in the catalysis, we produced Tip60-K327R and Tip60-K327Q mutants by using the site-directed mutagenesis protocol. It is well known that arginine is often introduced as a mimetic substitute of unacetylated lysine and glutamine as an acetylated lysine mimic. Thus, it is likely that Tip60-K327R mimics Tip60 with K327 in the unacetylated form and Tip60-K327Q mimics Tip60 with K327 in the acetylated form. First, we examined how K327 mutation affects the acetylation of the cognate substrate H4. Fig. 4 shows that the HAT activity of both Tip60-K327R and Tip60-K327Q mutants dramatically decreased; especially K327R mutation almost completely abolished the HAT activity. This result demonstrates that Tip60 catalysis is highly sensitive to the alternations on K327. It is noteworthy to point out that the HAT activity of Tip60-K327Q is 6-fold higher than Tip60-K327R, with 2.5% and 0.4% retained activity of the wt-Tip60 activity, respectively. These data implicate that Tip60 with unacetylated K327 might be catalytically inactive and acetylation at K327 promotes the enzymatic activity of Tip60. The reason for the weak activity of Tip60-K327Q in comparison to that of wt-Tip60 possibly may reflect that glutamine is not a precise mimic of the acetylated K327.

**Figure 5:**
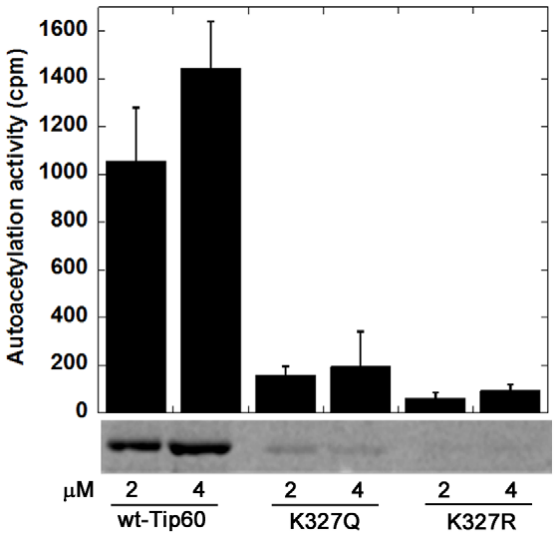
Effect of K327 mutation on the autoacetylation activity of Tip60. Each protein was tested at 2 and 4 µM, reacting with 30 µM [^14^C]-AcCoA for 30 min at 30°C.

**Figure 6:**
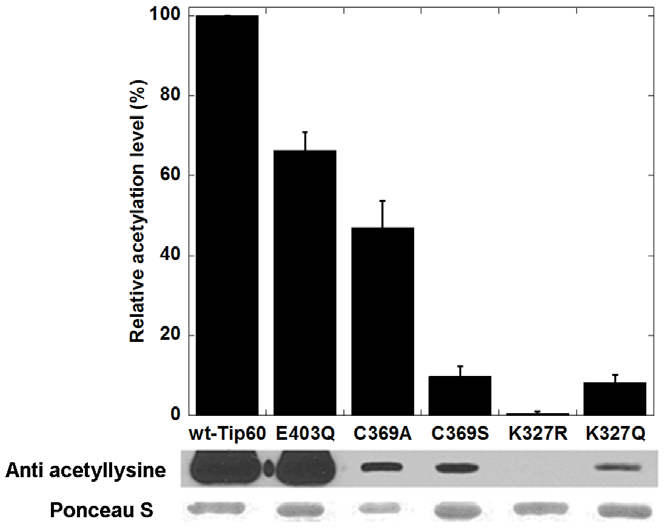
Levels of Tip60 autoacetylation in wt-Tip60 and mutant Tip60 examined by Western blot.

As aforementioned, Tip60 acetylates itself at several lysine residues in addition to K327. To test the effect of K327 mutation on Tip60 autoacetylation at other lysine sites, we reacted the three forms of Tip60 with [^14^C]-AcCoA and analyzed the protein autoacetylation activity by phosphorimaging. As seen on Fig. 5, the autoacetylation of Tip60-K327R was at the background level. On the other hand, although weaker than wt-Tip60, the autoacetylation of Tip60-K327Q was still clearly detectable. Because autoacetylation of recombinant Tip60 occurred during the period of protein expression in *E. coli*, we performed Western blot analysis using anti-acetyllysine antibody to quantify the acetylated lysine levels in the wt-Tip60 and Tip60-K327R and Tip60-K327Q mutants (Fig. 6). As expected, wt-Tip60 presented strong autoacetylation level. K327Q mutation decreased but not diminished the level of autoacetylation. In contrast, K327R mutation did not show any detectable autoacetylation. These results are in good agreement with the data about the H4 acetylation activity of Tip60 shown above.

### The effect of K327 deacetylation on the enzymatic activity of Tip60

**Figure 7:**
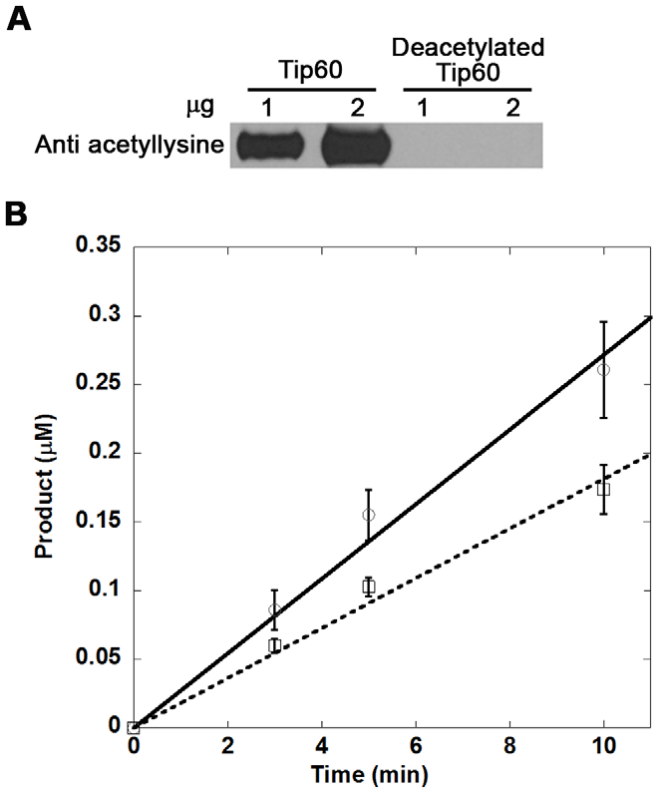
Deacetylation of Tip60 by Sirt1 and the activity of the deacetylated Tip60. (a) Tip60 was deactylated by Sirt1. (b) Deacetylated Tip60 showed weaker HAT activity. The HAT assays were carried out at 30°C. Each reaction contained 10 µM [^14^C]-AcCoA, 200 µM H4-20 and 0.02 µM Tip60 (solid line) or deacetylated Tip60 (dash line).

Although the mutational analysis of K327 is valuable to address the function of K327 acetylation in Tip60 catalysis, one potential pitfall is that the single site mutation may produce a detrimental effect on the enzyme local structures, thereby complicating the interpretation of the data. Also, Arg and Gln only mimic the structure of lysine residue to a limited degree in unacetylated and acetylated forms, respectively. A more unbiased approach would be to produce the Tip60 protein in the deacetylated form (Tip60^deac^) and thereby compare its catalytic activity with the acetylated Tip60 (Tip60^ac^). Sirt1 was reported to be a lysine deacetylase with promiscuous substrate specificity [54] and has been shown to deacetylate Tip60 [51]. Thus we carried out deacetylation of Tip60 with Sirt1 in the presence of NAD^+^. Following the deacetylation, Tip60 was purified by size exclusion chromatography. Western blot analysis using anti-acetyllysine antibody showed that Tip60 were nearly completely deacetylated after the treatment with Sirt1/NAD^+^ (Fig. 7A).

**Figure 8:**
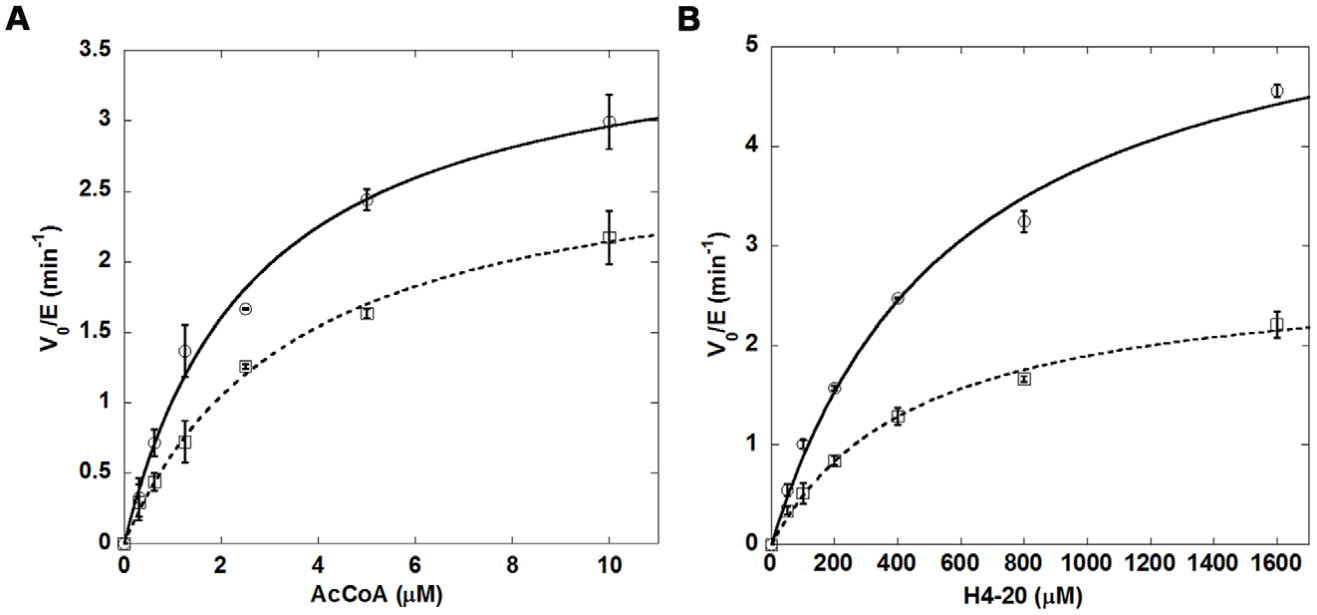
Substrate-dependent activity curves for Tip60^ac^ (solid line) and Tip60^deac^ (dash line). (a): 500 µM H4-20 and varied concentration of [^14^C]-AcCoA ranging from 0 to 10 µM. Enzyme concentration was 0.005 µM. (b): 10 µM [^14^C]-AcCoA and varied concentration of H4-20 ranging from 0 to 1600 µM. Enzyme concentration was 0.01 µM.

With Tip60^deac^ in hand, we studied and compared its cognate enzymatic activity with Tip60^ac^. The H4 acetylation reaction was carried out with 10 µM [^14^C]-AcCoA, 200 µM H4-20 and 20 nM enzyme at different reaction times. As depicted in Fig. 7B, Tip60^deac^ exhibited a decreased but still significant substrate acetylation activity compared with Tip60^ac^. This data gave strong indication that acetylation of K327 upregulates Tip60 activity; however, deacetylation at this site does not abrogate Tip60 activity. To corroborate this result, we measured the substrate dose-dependent curve of Tip60 activity. As shown in Fig. 8, both Tip60 in acetylated and deacetylated forms exhibited Michaelis-Menten like dose response curves. In comparison with Tip60^ac^, Tip60^deac^ contained decreased but still substantial amounts of activity at the selected substrate concentrations. By fitting the data to the Michaelis-Menten equation, we found that both *k_cat_* and *K_m_* values of Tip60^deac^ differ from that of Tip60^ac^, which suggests that acetylation of K327 affects the substrate binding affinity as well as the turnover rate of Tip60 catalysis.

### The role of cysteine-369 and glutamate-403 in Tip60 autoacetylation

**Figure 9:**
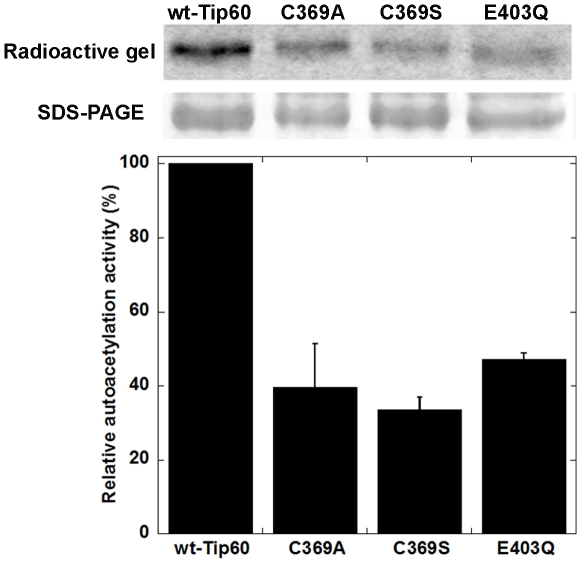
Effects of C369 and E403 mutations on the autoacetylation of Tip60 protein. In each reaction, 5 µM Tip60 protein was incubated with 10 µM [^14^C]-AcCoA for 1 h. The reaction was quenched by protein loading dye. The mixtures were solved on 12% SDS-PAGE, dried in vacuum, visualized by phosphorimaging, and analyzed by QuantityOne software.

One intriguing feature in the X-ray crystal structure of Tip60 HAT domain (PDB 2OU2) is that the sulfhydryl group of a cysteine residue, i.e. C369, is located in a position very close to the epsilon-nitrogen of K327, i.e. 3.9 Å. This unique spatial correlation leads us to postulate that C369 might play a critical role in the pathway of K327 acetylation. To investigate the role of C369 in Tip60 autoacetylation, we produced Tip60-C369A and Tip60-C369S mutants. In addition, E403 is another key residue in the active site and may play a general-base role for Tip60 catalysis, so we produced E403Q mutant using the site-directed mutagenesis method. All these Tip60 proteins were subjected to reaction with [^14^C]-AcCoA and the protein autoacetylation activity was examined by the phosphorimaging analysis. As shown in Fig. 9, the autoacetylation activities of all these Tip60 mutants decreased in comparison to that of wt-Tip60. Furthermore, we analyzed the autoacetylation level of these proteins using Western blot. Again, mutation of C369 and E403 decreased Tip60 autoacetylation (Fig. 6). These results support that these two residues are needed to maintain the full capacity of Tip60 autoacetylation. At this stage, we cannot distinguish whether the observed autoacetylation in C369A/S and E403Q mutants is solely due to K327 acetylation or also contributed by acetylation on the other lysine residues. Interestingly, The C369A mutant retains as much activity as C369S and E403Q mutants, suggesting that the cysteine sulfhydryl group is important, but not obligatory for Tip60 autoacetylation.

## Discussion

Our proteomic MS data showed that autoacetylation of Tip60 occurred in multiple lysine residues. In particular, K327 in Tip60 is a strictly conserved active site lysine residue in the MYST HATs. We showed that mutation of this active site lysine to arginine almost completely abrogated the catalytic activity of Tip60 in protein autoacetylation and H4 acetylation (Fig. 4 and 5). In contrast, mutation of lysine to glutamine (a possible acetyl-lysine mimic) still supported an appreciably higher activity, i.e. 2.5% of wt-Tip60 activity (Fig. 4). The strict conservation of K327 in other MYST proteins (e.g. K274 in MOF) suggests that autoacetylation at the active site lysine may be ubiquitous in this family of enzymes (Scheme 1). This argument is supported by several recent studies on MOF, another MYST protein. For instance, Sun et al. [55] showed that K274 in human MOF is in the acetylated state. Mutation of this lysine to Arg (K274R) almost completely abolishes hMOF activity (0.1%), while the K274Q mutant retained about 2.9% activity. This is very similar to the effect of K327 mutation that we observed for Tip60. In another study, K274 in drosophila MOF was also found in the acetylated state and mutation of this lysine to alanine caused a deleterious effect on the thermal stability of the protein and abrogated the catalytic activity [56]. Together, these findings based on site-directed mutagenesis highlight the significance of the active site autoacetylation for MYST catalysis. However, a limitation for the mutation method is that mutation of lysine to glutamine and arginine only moderately mimics the lysine in the acetylated and unacetylated form, respectively. Thus, caveat should be taken for the interpretation of the effect of autoacetylation from the mutational studies. In this study, we were able to produce Tip60 in the deacetylated form by treatment with Sirt1/NAD^+^. Enzymatic analysis with the deacetylated Tip60 showed that, in the deacetylated state, the enzymatic activity of Tip60 decreased but was not abolished. Therefore, we suggest that acetylation of K327 upregulates but is not obligatory for the cognate enzymatic activity of Tip60. Furthermore, we found that mutation of cysteine 369 to Ala or Ser decreased but not abrogated the autoacetylation activity (Fig. 6 and Fig. 9) and the cognate H4 acetylation activity (data not shown) of Tip60, highlighting that the sulfhydryl group on C369 is important, but is dispensable for Tip60 activity. Previous studies showed that lysine autoacetylation also occurs in the other HAT family proteins. For instance, autoacetylation of PCAF increases the nuclear localization of the protein [57]. HAT p300 contains a proteolytically sensitive autoactivation loop motif that harbors multiple lysine acetylation sites [58], [59]. Rtt109 catalyzes intramolecular autoacetylation at Lys-290, which is in a buried site and located in a helical segment [60], [61], [62]. Biochemical assays showed that Rtt109 autoacetylation stimulates HAT activity by increasing AcCoA binding affinity and by enhancing the rate of acetyl group transfer [63]. Thus, autoacetylation appears to be an important mechanism utilized by cells for HAT activity regulation. However, given the huge difference in the regions that are acetylated and the spatial orientation of the acetylated lysine residues relative to the active site, the mode and function of autoacetylation in regulating the activities of each HAT appear to be significantly distinct from one another. The active site lysine autoacetylation in Tip60 showcases another mechanism of HAT activity regulation in HAT biology. Additional studies are warranted to delineate in detail how the active site lysine autoacetylation in MYST HATs is regulated in various cellular signaling cascades. Furthermore, in addition to the active site lysine acetylation, Tip60 protein contains acetylated several lysine residues in other domain regions, e.g. Lys-76 and Lys-80 in the chromodomain. Further biochemical studies will be required to determine the mechanistic differences between these two classes of autoacetylation events in Tip60 activity modulation.

## Materials and Methods

### Site-directed mutagenesis of Tip60

Optimized DNA sequence encoding the full length Tip60 alpha protein was subcloned to bacterial expression vector pET21a (+). Site-directed mutagenesis for the production of Tip60 protein mutants was performed using the QuikChange procedure (Stratagene). All mutations were confirmed by DNA sequencing.

#### Protein expression and purification

Each Tip60 DNA plasmid or pDEST-Sirt1 plasmid (gift from Dr. Lin at Cornell University) was introduced into BL21 (DE3) using the heat shock transformation method. Protein expression was induced with 0.3 mM IPTG at 16°C for 20 h. After protein expression, cells were pelleted by centrifuge; suspended in the lysis buffer (25 mM HEPES pH 8.0, 500 mM NaCl, 1 mM PMSF, 1 mM MgSO4, 5% ethylene glycol, 10% glycerol) and lysed by French Press. The protein supernatant was purified on the Ni-charged His6x-tag binding resin (Novagen). Before protein loading, the beads were equilibrated with a column buffer (25 mM Na-HEPES, pH 7.0, 300 mM NaCl, 1 mM PMSF, 10% glycerol and 30 mM imidazole). After protein loading, the beads were washed thoroughly with the column buffer (25 mM Na-HEPES, pH 7.0, 300 mM NaCl, 1 mM PMSF, 10% glycerol and 70 mM imidazole), and then the protein was eluted with the elution buffer (25 mM Na-HEPES, pH 7.0, 300 mM NaCl, 1 mM PMSF, 100 mM EDTA, 10% glycerol and 200 mM imidazole). Different elution fractions were individually checked on SDS–PAGE. The pure protein fractions were combined and dialyzed against the storage buffer (25 mM Na-HEPES, pH 7.0, 500 mM NaCl, 1 mM EDTA, 1 mM DTT and 10% glycerol) at 4°C. After dialysis, protein solutions were concentrated using Millipore centrifugal filters. Concentrations of protein were measured using the Bradford assay. The final protein samples were aliquoted, flash-frozen, and stored in the −80°C freezer.

#### MS/MS analysis of Tip60 acetylation

Recombinant full length Tip60 protein was digested with AspN, LysC, and Trypsin, and run as LC-MS/MS samples. These results were all searched with the same parameters, which included variable modifications on methionine (oxidation), lysine (methyl, acetyl), and cysteine (IAA). The trypsin digests were processed by Mascot with a partial digestion setting, and the other two enzyme digests were processed with SEQUEST and further filtered using the transproteomic pipeline software. All data sets were combined in SCAFFOLD and the output file was produced.

#### Acetylation assays

Both Tip60 autoacetylation and histone acetylation assays were carried out at 30°C in a reaction buffer containing 50 mM HEPES (pH 8.0), 0.1 mM EDTA, and 1 mM DTT. Typically, [^14^C]-labeled acetyl-CoA (Perkin Elmer) was used as the acetyl donor. After the acetylation reaction, the mixture was resolved on SDS-PAGE. The gel was dried in vacuum and then exposed to a phosphor screen (GE Healthcare). Autoradiograph was scanned on a Typhoon scanner and analyzed by QuantityOne software. For the acetylation of histone H4, a synthetic peptide corresponding to the first 20 amino acid sequence of the histone H4 N-terminal tail, i.e. H4-20 (ac-SGRGKGGKGLGKGGAKRHRK), was used as the substrate. In the filter binding assay for measuring H4-20 acetylation, the reaction was quenched by spotting the reaction mixture on a P81 filter paper disc (Whatman). After the paper discs were washed with 50 mM NaHCO_3_ (pH 9.0) and air dried, liquid scintillation counting was performed to measure the amount of acetylated products. For the Western blot analysis of Tip60 autoacetylation, anti-acetyl lysine antibody (Calbiochem, ST1027) was used as the primary antibody and goat anti-rabbit IgG-HRP (Santa Cruz Biotechnology, sc-2004) as the secondary antibody.

#### Preparation of Deacetylated Tip60 (Tip60^ac^)

Deacetylation of Tip60 was carried out in 500 µL reaction mixture containing 50 mM HEPES, pH 8.0, 1 mM DTT, 17.5 µM Sirt1, 9 mM NAD^+^ and 12 µM Tip60 at 25°C for 1.5 h. Separation of deacetylated Tip60 was accomplished by using size exclusion chromatography in the column buffer containing 25 mM HEPES, pH 7.0, 200 mM NaCl, 5% Glycerol and 1 mM DTT. The target elute was collected and concentrated by Millipore centrifugal filters. Concentration of protein was measured by Bradford assay. The final protein samples were aliquoted, flash-frozen, and stored at −80°C. The control of Tip60 was prepared with the same condition in the absence of Sirt1.

## Supporting Information

Supporting Information S1 **Figures S1 through S11 show the tandem mass spectrometric data for the acetylated lysine residues in Tip60 protein.** Figure S1. LC-MS/MS analysis of the AA(67-80) peptide sequence showing Tip60 is acetylated at lysine 76. Figure S2. LC-MS/MS analysis of the AA(77-104) peptide sequence showing Tip60 is acetylated at lysine 80. Figure S3. LC-MS/MS analysis of the AA(98-120) peptide sequence showing Tip60 is acetylated at lysine 104. Figure S4. LC-MS/MS analysis of the AA(94-115) peptide sequence showing Tip60 is acetylated at lysine 104. Figure S5. LC-MS/MS analysis of the AA(94-124) peptide sequence showing Tip60 is acetylated at lysine 104. Figure S6. LC-MS/MS analysis of the AA(150-177) peptide sequence showing Tip60 is acetylated at lysine 150. Figure S7. LC-MS/MS analysis of the AA(180-188) peptide sequence showing Tip60 is acetylated at lysine 187. Figure S8. LC-MS/MS analysis of the AA(324-347) peptide sequence showing Tip60 is acetylated at lysine 327. Figure S9. LC-MS/MS analysis of the AA(320-334) peptide sequence showing Tip60 is acetylated at lysine 327. Figure S10. LC-MS/MS analysis of the AA(320-340) peptide sequence showing Tip60 is acetylated at lysine 327. Figure S11. LC-MS/MS analysis of the AA(380-398) peptide sequence showing Tip60 is acetylated at lysine 383.
(PDF)
