# Supplementary material for: Function of the Active Site Lysine Autoacetylation in Tip60 Catalysis
Source: PLoS One. 2012 Mar 28;7(3):e32886. doi: 10.1371/journal.pone.0032886 (PMC3314657; doi:10.1371/journal.pone.0032886)
Supplement: Supporting Information S1 — Figures S1 through S11 show the tandem mass spectrometric data for the acetylated lysine residues in Tip60 protein. Figure S1. LC-MS/MS analysis of the AA(67-80) peptide sequence showing Tip60 is acetylated at lysine 76. Figure S2. LC-MS/MS analysis of the AA(77-104) peptide sequence showing Tip60 is acetylated at lysine 80. Figure S3. LC-MS/MS analysis of the AA(98-120) peptide sequence showing Tip60 is acetylated at lysine 104. Figure S4. LC-MS/MS analysis of the AA(94-115) peptide sequence showing Tip60 is acetylated at lysine 104. Figure S5. LC-MS/MS analysis of the AA(94-124) peptide sequence showing Tip60 is acetylated at lysine 104. Figure S6. LC-MS/MS analysis of the AA(150-177) peptide sequence showing Tip60 is acetylated at lysine 150. Figure S7. LC-MS/MS analysis of the AA(180-188) peptide sequence showing Tip60 is acetylated at lysine 187. Figure S8. LC-MS/MS analysis of the AA(324-347) peptide sequence showing Tip60 is acetylated at lysine 327. Figure S9. LC-MS/MS analysis of the AA(320-334) peptide sequence showing Tip60 is acetylated at lysine 327. Figure S10. LC-MS/MS analysis of the AA(320-340) peptide sequence showing Tip60 is acetylated at lysine 327. Figure S11. LC-MS/MS analysis of the AA(380-398) peptide sequence showing Tip60 is acetylated at lysine 383. (PDF) [file pone.0032886.s001.pdf]

C. Yang, et al.

Supporting Information

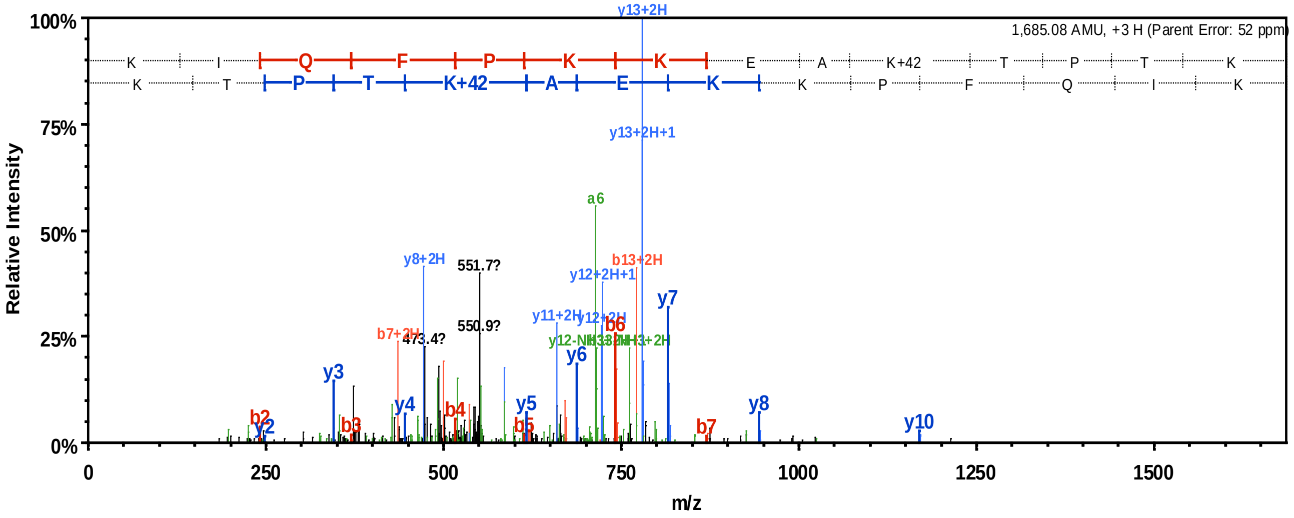

B	B Ions	B+2H	B-NH3	B-H2O	AA	Y Ions	Y+2H	Y-NH3	Y-H2O	Y
1	129.1	65.1	112.1		K	1,686.0	843.5	1,669.0	1,668.0	14
2	242.2	121.6	225.2		I	1,557.9	779.5	1,540.9	1,539.9	13
3	370.2	185.6	353.2		Q	1,444.8	722.9	1,427.8	1,426.8	12
4	517.3	259.2	500.3		F	1,316.8	658.9	1,299.7	1,298.7	11
5	614.4	307.7	597.3		P	1,169.7	585.3	1,152.7	1,151.7	10
6	742.5	371.7	725.4		K	1,072.6	536.8	1,055.6	1,054.6	9
7	870.6	435.8	853.5		K	944.5	472.8	927.5	926.5	8
8	999.6	500.3	982.6	981.6	E	816.4	408.7	799.4	798.4	7
9	1,070.6	535.8	1,053.6	1,052.6	A	687.4	344.2	670.4	669.4	6
10	1,240.7	620.9	1,223.7	1,222.7	K+42	616.4	308.7	599.3	598.4	5
11	1,341.8	671.4	1,324.8	1,323.8	T	446.3		429.2	428.3	4
12	1,438.8	719.9	1,421.8	1,420.8	P	345.2		328.2	327.2	3
13	1,539.9	770.4	1,522.9	1,521.9	T	248.2		231.1	230.1	2
14	1,686.0	843.5	1,669.0	1,668.0	K	147.1		130.1		1

Figure S1. LC-MS/MS analysis of the AA(67-80) peptide sequence showing Tip60 is acetylated at lysine 76.

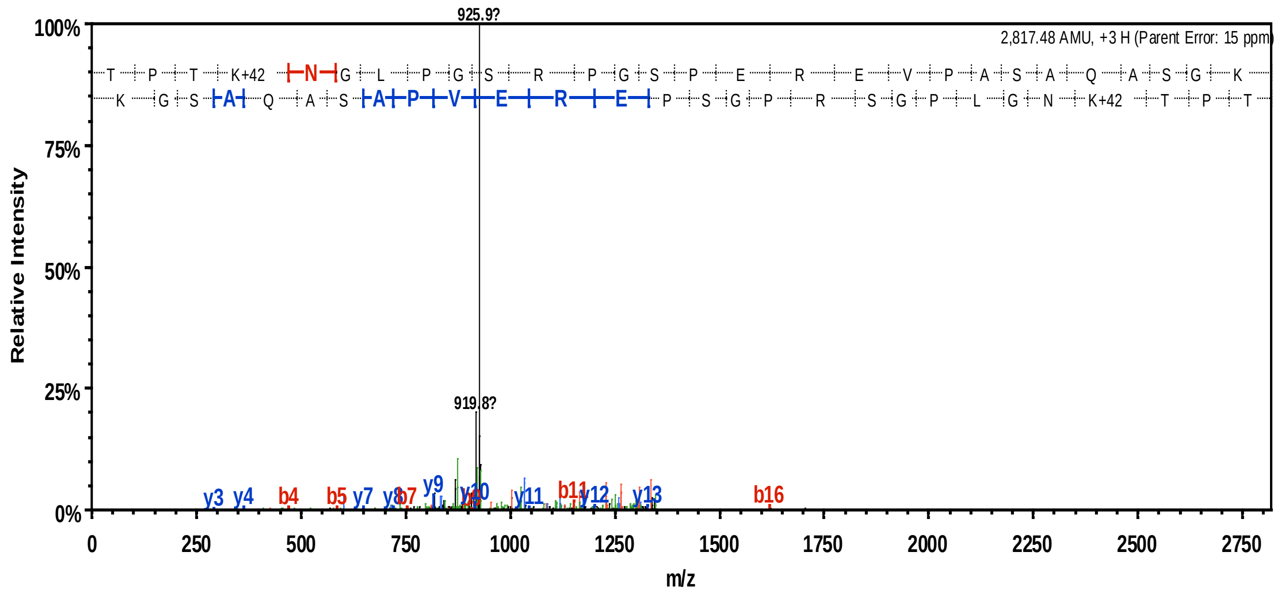

B	B Ions	B+2H	B-NH3	B-H2O	AA	Y Ions	Y+2H	Y-NH3	Y-H2O	Y
1	102.1			84.0	T	2,818.4	1,409.7	2,801.4	2,800.4	28
2	199.1			181.1	P	2,717.4	1,359.2	2,700.4	2,699.4	27
3	300.2			282.1	T	2,620.3	1,310.7	2,603.3	2,602.3	26
4	470.3	235.6	453.2	452.3	K+42	2,519.3	1,260.2	2,502.3	2,501.3	25
5	584.3	292.7	567.3	566.3	N	2,349.2	1,175.1	2,332.2	2,331.2	24
6	641.3	321.2	624.3	623.3	G	2,235.1	1,118.1	2,218.1	2,217.1	23
7	754.4	377.7	737.4	736.4	L	2,178.1	1,089.6	2,161.1	2,160.1	22
8	851.5	426.2	834.4	833.5	P	2,065.0	1,033.0	2,048.0	2,047.0	21
9	908.5	454.7	891.5	890.5	G	1,968.0	984.5	1,951.0	1,950.0	20
10	995.5	498.3	978.5	977.5	S	1,911.0	956.0	1,893.9	1,893.0	19
11	1,151.6	576.3	1,134.6	1,133.6	R	1,823.9	912.5	1,806.9	1,805.9	18
12	1,248.7	624.8	1,231.6	1,230.7	P	1,667.8	834.4	1,650.8	1,649.8	17
13	1,305.7	653.3	1,288.7	1,287.7	G	1,570.8	785.9	1,553.8	1,552.8	16
14	1,392.7	696.9	1,375.7	1,374.7	S	1,513.8	757.4	1,496.7	1,495.7	15
15	1,489.8	745.4	1,472.7	1,471.8	P	1,426.7	713.9	1,409.7	1,408.7	14
16	1,618.8	809.9	1,601.8	1,600.8	E	1,329.7	665.3	1,312.6	1,311.7	13
17	1,774.9	888.0	1,757.9	1,756.9	R	1,200.6	600.8	1,183.6	1,182.6	12
18	1,904.0	952.5	1,886.9	1,886.0	E	1,044.5	522.8	1,027.5	1,026.5	11
19	2,003.0	1,002.0	1,986.0	1,985.0	V	915.5	458.2	898.5	897.5	10
20	2,100.1	1,050.5	2,083.1	2,082.1	P	816.4	408.7	799.4	798.4	9
21	2,171.1	1,086.1	2,154.1	2,153.1	A	719.4	360.2	702.3	701.4	8
22	2,258.2	1,129.6	2,241.1	2,240.1	S	648.3	324.7	631.3	630.3	7
23	2,329.2	1,165.1	2,312.2	2,311.2	A	561.3	281.2	544.3	543.3	6
24	2,457.2	1,229.1	2,440.2	2,439.2	Q	490.3		473.2	472.3	5
25	2,528.3	1,264.6	2,511.3	2,510.3	A	362.2		345.2	344.2	4
26	2,615.3	1,308.2	2,598.3	2,597.3	S	291.2		274.1	273.2	3
27	2,672.3	1,336.7	2,655.3	2,654.3	G	204.1		187.1		2
28	2,818.4	1,409.7	2,801.4	2,800.4	K	147.1		130.1		1

Figure S2. LC-MS/MS analysis of the AA(77-104) peptide sequence showing Tip60 is acetylated at lysine 80.

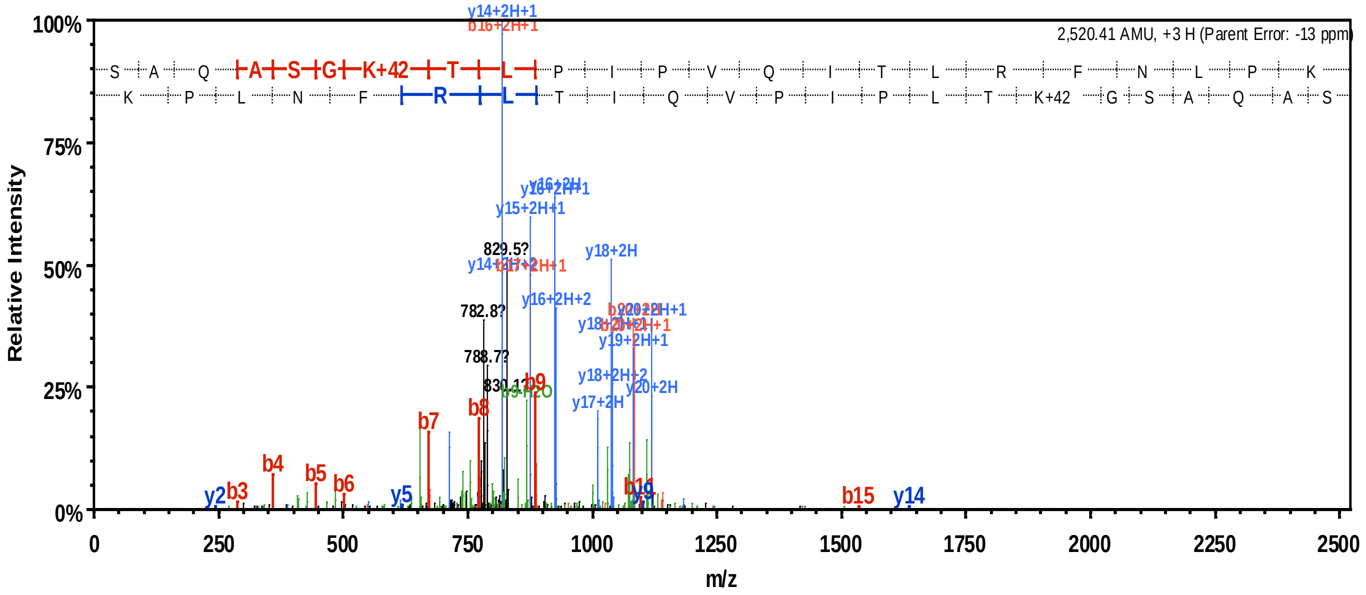

B	B Ions	B+2H	B-NH3	B-H2O	AA	Y Ions	Y+2H	Y-NH3	Y-H2O	Y
1	88.0			70.0	S	2,521.5	1,261.2	2,504.4	2,503.4	23
2	159.1			141.1	A	2,434.4	1,217.7	2,417.4	2,416.4	22
3	287.1		270.1	269.1	Q	2,363.4	1,182.2	2,346.4	2,345.4	21
4	358.2		341.1	340.2	A	2,235.3	1,118.2	2,218.3	2,217.3	20
5	445.2		428.2	427.2	S	2,164.3	1,082.6	2,147.3	2,146.3	19
6	502.2	251.6	485.2	484.2	G	2,077.3	1,039.1	2,060.2	2,059.2	18
7	672.3	336.7	655.3	654.3	K+42	2,020.2	1,010.6	2,003.2	2,002.2	17
8	773.4	387.2	756.4	755.4	T	1,850.1	925.6	1,833.1	1,832.1	16
9	886.5	443.7	869.4	868.5	L	1,749.1	875.0	1,732.1	1,731.1	15
10	983.5	492.3	966.5	965.5	P	1,636.0	818.5	1,619.0	1,618.0	14
11	1,096.6	548.8	1,079.6	1,078.6	I	1,538.9	770.0	1,521.9	1,520.9	13
12	1,193.7	597.3	1,176.6	1,175.6	P	1,425.9	713.4	1,408.8	1,407.8	12
13	1,292.7	646.9	1,275.7	1,274.7	V	1,328.8	664.9	1,311.8	1,310.8	11
14	1,420.8	710.9	1,403.8	1,402.8	Q	1,229.7	615.4	1,212.7	1,211.7	10
15	1,533.9	767.4	1,516.8	1,515.9	I	1,101.7	551.3	1,084.7	1,083.7	9
16	1,634.9	818.0	1,617.9	1,616.9	T	988.6	494.8	971.6	970.6	8
17	1,748.0	874.5	1,731.0	1,730.0	L	887.5	444.3	870.5		7
18	1,904.1	952.6	1,887.1	1,886.1	R	774.5	387.7	757.4		6
19	2,051.2	1,026.1	2,034.1	2,033.2	F	618.4		601.3		5
20	2,165.2	1,083.1	2,148.2	2,147.2	N	471.3		454.3		4
21	2,278.3	1,139.6	2,261.3	2,260.3	L	357.2		340.2		3
22	2,375.3	1,188.2	2,358.3	2,357.3	P	244.2		227.1		2
23	2,521.5	1,261.2	2,504.4	2,503.4	K	147.1		130.1		1

Figure S3. LC-MS/MS analysis of the AA(98-120) peptide sequence showing Tip60 is acetylated at lysine 104.

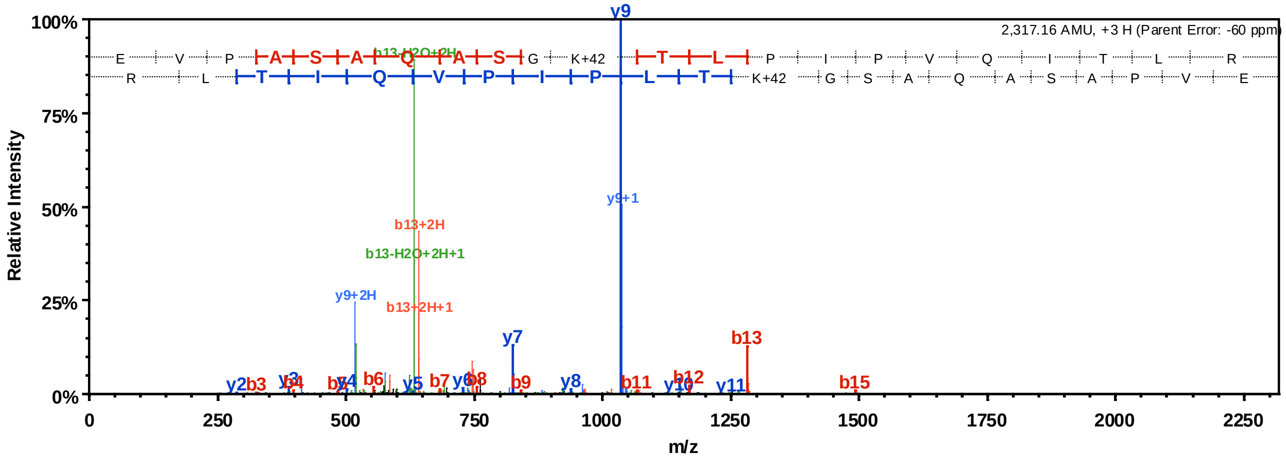

B	B Ions	B+2H	B-NH3	B-H2O	AA	Y Ions	Y+2H	Y-NH3	Y-H2O	Y
1	130.0			112.0	E	2,318.3	1,159.7	2,301.3	2,300.3	22
2	229.1			211.1	V	2,189.3	1,095.1	2,172.2	2,171.3	21
3	326.2			308.2	P	2,090.2	1,045.6	2,073.2	2,072.2	20
4	397.2			379.2	A	1,993.1	997.1	1,976.1	1,975.1	19
5	484.2			466.2	S	1,922.1	961.6	1,905.1	1,904.1	18
6	555.3	278.1		537.3	A	1,835.1	918.0	1,818.0	1,817.1	17
7	683.3	342.2	666.3	665.3	Q	1,764.0	882.5	1,747.0	1,746.0	16
8	754.4	377.7	737.3	736.4	A	1,636.0	818.5	1,619.0	1,618.0	15
9	841.4	421.2	824.4	823.4	S	1,564.9	783.0	1,547.9	1,546.9	14
10	898.4	449.7	881.4	880.4	G	1,477.9	739.5	1,460.9	1,459.9	13
11	1,068.5	534.8	1,051.5	1,050.5	K+42	1,420.9	710.9	1,403.9	1,402.9	12
12	1,169.6	585.3	1,152.6	1,151.6	T	1,250.8	625.9	1,233.8	1,232.8	11
13	1,282.7	641.8	1,265.6	1,264.7	L	1,149.7	575.4	1,132.7	1,131.7	10
14	1,379.7	690.4	1,362.7	1,361.7	P	1,036.7	518.8	1,019.6	1,018.6	9
15	1,492.8	746.9	1,475.8	1,474.8	I	939.6	470.3	922.6	921.6	8
16	1,589.9	795.4	1,572.8	1,571.8	P	826.5	413.8	809.5	808.5	7
17	1,688.9	845.0	1,671.9	1,670.9	V	729.5	365.2	712.4	711.5	6
18	1,817.0	909.0	1,800.0	1,799.0	Q	630.4		613.4	612.4	5
19	1,930.1	965.5	1,913.0	1,912.1	I	502.3		485.3	484.3	4
20	2,031.1	1,016.1	2,014.1	2,013.1	T	389.3		372.2	371.2	3
21	2,144.2	1,072.6	2,127.2	2,126.2	L	288.2		271.2		2
22	2,318.3	1,159.7	2,301.3	2,300.3	R	175.1		158.1		1

Figure S4. LC-MS/MS analysis of the AA(94-115) peptide sequence showing Tip60 is acetylated at lysine 104.

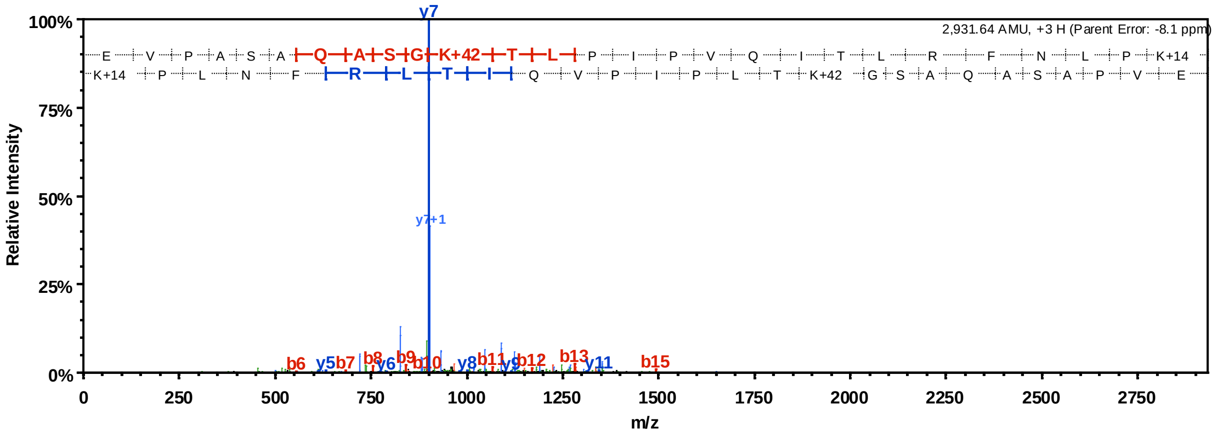

B	B Ions	B+2H	B-NH3	B-H2O	AA	Y Ions	Y+2H	Y-NH3	Y-H2O	Y
1	130.0			112.0	E	2,931.7	1,466.3	2,914.6	2,913.7	27
2	229.1			211.1	V	2,802.6	1,401.8	2,785.6	2,784.6	26
3	326.2			308.2	P	2,703.6	1,352.3	2,686.5	2,685.5	25
4	397.2			379.2	A	2,606.5	1,303.8	2,589.5	2,588.5	24
5	484.2			466.2	S	2,535.5	1,268.2	2,518.4	2,517.5	23
6	555.3	278.1		537.3	A	2,448.4	1,224.7	2,431.4	2,430.4	22
7	683.3	342.2	666.3	665.3	Q	2,377.4	1,189.2	2,360.4	2,359.4	21
8	754.4	377.7	737.3	736.4	A	2,249.3	1,125.2	2,232.3	2,231.3	20
9	841.4	421.2	824.4	823.4	S	2,178.3	1,089.7	2,161.3	2,160.3	19
10	898.4	449.7	881.4	880.4	G	2,091.3	1,046.1	2,074.2	2,073.3	18
11	1,068.5	534.8	1,051.5	1,050.5	K+42	2,034.2	1,017.6	2,017.2	2,016.2	17
12	1,169.6	585.3	1,152.6	1,151.6	T	1,864.1	932.6	1,847.1	1,846.1	16
13	1,282.7	641.8	1,265.6	1,264.7	L	1,763.1	882.1	1,746.1	1,745.1	15
14	1,379.7	690.4	1,362.7	1,361.7	P	1,650.0	825.5	1,633.0	1,632.0	14
15	1,492.8	746.9	1,475.8	1,474.8	I	1,553.0	777.0	1,535.9	1,534.9	13
16	1,589.9	795.4	1,572.8	1,571.8	P	1,439.9	720.4	1,422.8	1,421.9	12
17	1,688.9	845.0	1,671.9	1,670.9	V	1,342.8	671.9	1,325.8	1,324.8	11
18	1,817.0	909.0	1,800.0	1,799.0	Q	1,243.8	622.4	1,226.7	1,225.7	10
19	1,930.1	965.5	1,913.0	1,912.1	I	1,115.7	558.4	1,098.7	1,097.7	9
20	2,031.1	1,016.1	2,014.1	2,013.1	T	1,002.6	501.8	985.6	984.6	8
21	2,144.2	1,072.6	2,127.2	2,126.2	L	901.6	451.3	884.5		7
22	2,300.3	1,150.7	2,283.3	2,282.3	R	788.5	394.7	771.5		6
23	2,447.4	1,224.2	2,430.3	2,429.4	F	632.4		615.4		5
24	2,561.4	1,281.2	2,544.4	2,543.4	N	485.3		468.3		4
25	2,674.5	1,337.8	2,657.5	2,656.5	L	371.3		354.2		3
26	2,771.5	1,386.3	2,754.5	2,753.5	P	258.2		241.2		2
27	2,931.7	1,466.3	2,914.6	2,913.7	K+14	161.1		144.1		1

Figure S5. LC-MS/MS analysis of the AA(94-124) peptide sequence showing Tip60 is acetylated at lysine 104.

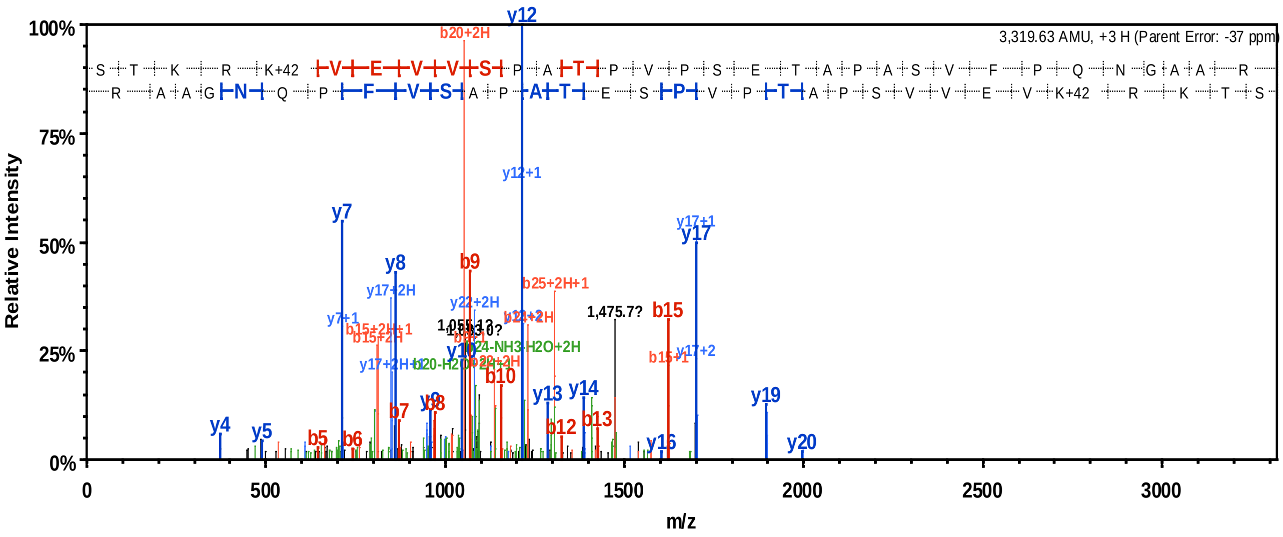

B	B Ions	B+2H	B-NH3	B-H2O	AA	Y Ions	Y+2H	Y-NH3	Y-H2O	Y
1	88.0			70.0	S	3,320.8	1,660.9	3,303.7	3,302.7	32
2	189.1			171.1	T	3,233.7	1,617.4	3,216.7	3,215.7	31
3	317.2	159.1	300.2	299.2	K	3,132.7	1,566.8	3,115.7	3,114.7	30
4	473.3	237.1	456.3	455.3	R	3,004.6	1,502.8	2,987.6	2,986.6	29
5	643.4	322.2	626.4	625.4	K+42	2,848.5	1,424.7	2,831.5	2,830.5	28
6	742.5	371.7	725.4	724.4	V	2,678.4	1,339.7	2,661.4	2,660.4	27
7	871.5	436.3	854.5	853.5	E	2,579.3	1,290.2	2,562.3	2,561.3	26
8	970.6	485.8	953.5	952.6	V	2,450.3	1,225.6	2,433.2	2,432.3	25
9	1,069.6	535.3	1,052.6	1,051.6	V	2,351.2	1,176.1	2,334.2	2,333.2	24
10	1,156.7	578.8	1,139.6	1,138.7	S	2,252.1	1,126.6	2,235.1	2,234.1	23
11	1,253.7	627.4	1,236.7	1,235.7	P	2,165.1	1,083.1	2,148.1	2,147.1	22
12	1,324.8	662.9	1,307.7	1,306.7	A	2,068.0	1,034.5	2,051.0	2,050.0	21
13	1,425.8	713.4	1,408.8	1,407.8	T	1,997.0	999.0	1,980.0	1,979.0	20
14	1,522.9	761.9	1,505.8	1,504.8	P	1,896.0	948.5	1,878.9	1,878.0	19
15	1,621.9	811.5	1,604.9	1,603.9	V	1,798.9	900.0	1,781.9	1,780.9	18
16	1,719.0	860.0	1,702.0	1,701.0	P	1,699.8	850.4	1,682.8	1,681.8	17
17	1,806.0	903.5	1,789.0	1,788.0	S	1,602.8	801.9	1,585.8	1,584.8	16
18	1,935.1	968.0	1,918.0	1,917.0	E	1,515.8	758.4	1,498.7	1,497.7	15
19	2,036.1	1,018.6	2,019.1	2,018.1	T	1,386.7	693.9	1,369.7	1,368.7	14
20	2,107.1	1,054.1	2,090.1	2,089.1	A	1,285.7	643.3	1,268.6	1,267.7	13
21	2,204.2	1,102.6	2,187.2	2,186.2	P	1,214.6	607.8	1,197.6	1,196.6	12
22	2,275.2	1,138.1	2,258.2	2,257.2	A	1,117.6	559.3	1,100.5	1,099.6	11
23	2,362.3	1,181.6	2,345.2	2,344.3	S	1,046.5	523.8	1,029.5	1,028.5	10
24	2,461.3	1,231.2	2,444.3	2,443.3	V	959.5	480.3	942.5		9
25	2,608.4	1,304.7	2,591.4	2,590.4	F	860.4	430.7	843.4		8
26	2,705.5	1,353.2	2,688.4	2,687.4	P	713.4	357.2	696.3		7
27	2,833.5	1,417.3	2,816.5	2,815.5	Q	616.3	308.7	599.3		6
28	2,947.6	1,474.3	2,930.5	2,929.5	N	488.3		471.2		5
29	3,004.6	1,502.8	2,987.5	2,986.6	G	374.2		357.2		4
30	3,075.6	1,538.3	3,058.6	3,057.6	A	317.2		300.2		3
31	3,146.6	1,573.8	3,129.6	3,128.6	A	246.2		229.1		2
32	3,320.8	1,660.9	3,303.7	3,302.7	R	175.1		158.1		1

Figure S6. LC-MS/MS analysis of the AA(150-177) peptide sequence showing Tip60 is acetylated at lysine 150.

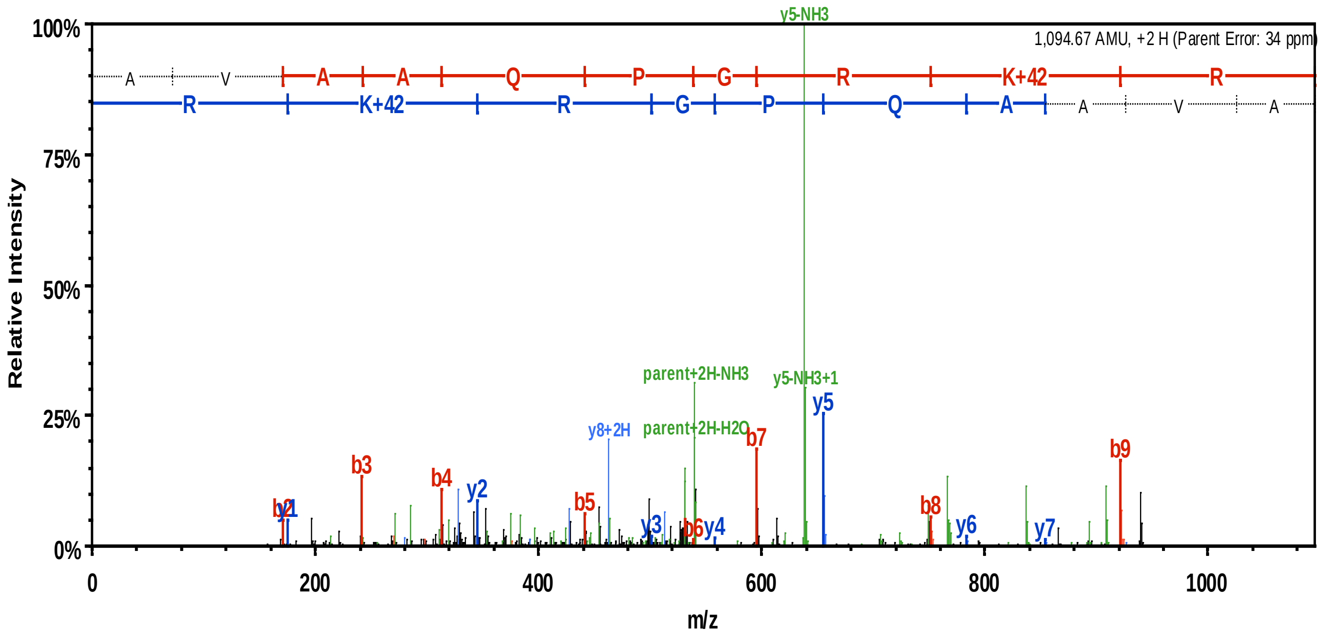

B	B Ions	B+2H	B-NH3	B-H2O	AA	Y Ions	Y+2H	Y-NH3	Y-H2O	Y
1	72.0				A	1,095.6	548.3	1,078.6		10
2	171.1				V	1,024.6	512.8	1,007.6		9
3	242.1				A	925.5	463.3	908.5		8
4	313.2				A	854.5	427.8	837.5		7
5	441.2		424.2		Q	783.5	392.2	766.4		6
6	538.3	269.7	521.3		P	655.4	328.2	638.4		5
7	595.3	298.2	578.3		G	558.3	279.7	541.3		4
8	751.4	376.2	734.4		R	501.3	251.2	484.3		3
9	921.5	461.3	904.5		K+42	345.2	173.1	328.2		2
10	1,095.6	548.3	1,078.6		R	175.1		158.1		1

Figure S7. LC-MS/MS analysis of the AA(180-188) peptide sequence showing Tip60 is acetylated at lysine 187.

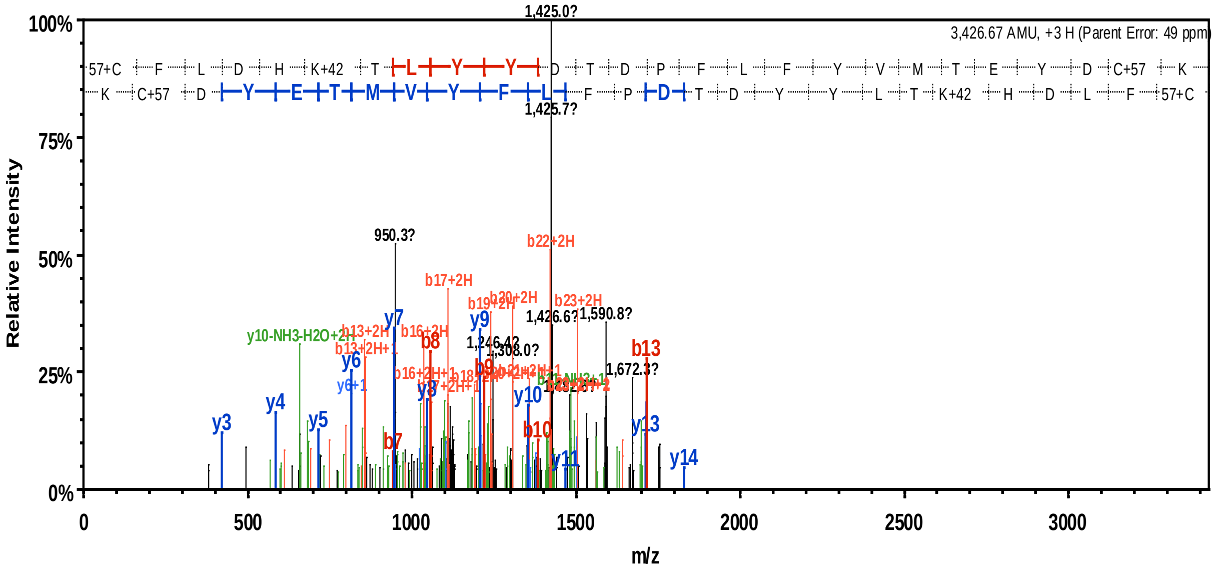

B	B Ions	B+2H	B-NH3	B-H2O	AA	Y Ions	Y+2H	Y-NH3	Y-H2O	Y
1	161.0				C+57	3,426.5	1,713.8	3,409.5	3,408.5	26
2	308.1				F	3,266.5	1,633.7	3,249.4	3,248.5	25
3	421.2				L	3,119.4	1,560.2	3,102.4	3,101.4	24
4	536.2			518.2	D	3,006.3	1,503.7	2,989.3	2,988.3	23
5	673.3	337.1		655.3	H	2,891.3	1,446.2	2,874.3	2,873.3	22
6	843.4	422.2	826.4	825.4	K+42	2,754.2	1,377.6	2,737.2	2,736.2	21
7	944.4	472.7	927.4	926.4	T	2,584.1	1,292.6	2,567.1	2,566.1	20
8	1,057.5	529.3	1,040.5	1,039.5	L	2,483.1	1,242.0	2,466.1	2,465.1	19
9	1,220.6	610.8	1,203.6	1,202.6	Y	2,370.0	1,185.5	2,353.0	2,352.0	18
10	1,383.6	692.3	1,366.6	1,365.6	Y	2,206.9	1,104.0	2,189.9	2,188.9	17
11	1,498.7	749.8	1,481.6	1,480.7	D	2,043.9	1,022.4	2,026.8	2,025.9	16
12	1,599.7	800.4	1,582.7	1,581.7	T	1,928.8	964.9	1,911.8	1,910.8	15
13	1,714.7	857.9	1,697.7	1,696.7	D	1,827.8	914.4	1,810.8	1,809.8	14
14	1,811.8	906.4	1,794.8	1,793.8	P	1,712.8	856.9	1,695.7	1,694.8	13
15	1,958.9	979.9	1,941.8	1,940.9	F	1,615.7	808.4	1,598.7	1,597.7	12
16	2,071.9	1,036.5	2,054.9	2,053.9	L	1,468.6	734.8	1,451.6	1,450.6	11
17	2,219.0	1,110.0	2,202.0	2,201.0	F	1,355.6	678.3	1,338.5	1,337.6	10
18	2,382.1	1,191.5	2,365.1	2,364.1	Y	1,208.5	604.8	1,191.5	1,190.5	9
19	2,481.1	1,241.1	2,464.1	2,463.1	V	1,045.4	523.2	1,028.4	1,027.4	8
20	2,612.2	1,306.6	2,595.2	2,594.2	M	946.4	473.7	929.3	928.4	7
21	2,713.2	1,357.1	2,696.2	2,695.2	T	815.3	408.2	798.3	797.3	6
22	2,842.3	1,421.6	2,825.3	2,824.3	E	714.3		697.2	696.3	5
23	3,005.3	1,503.2	2,988.3	2,987.3	Y	585.2		568.2	567.2	4
24	3,120.4	1,560.7	3,103.3	3,102.4	D	422.2		405.1	404.2	3
25	3,280.4	1,640.7	3,263.4	3,262.4	C+57	307.1		290.1		2
26	3,426.5	1,713.8	3,409.5	3,408.5	K	147.1		130.1		1

Figure S8. LC-MS/MS analysis of the AA(324-347) peptide sequence showing Tip60 is acetylated at lysine 327.

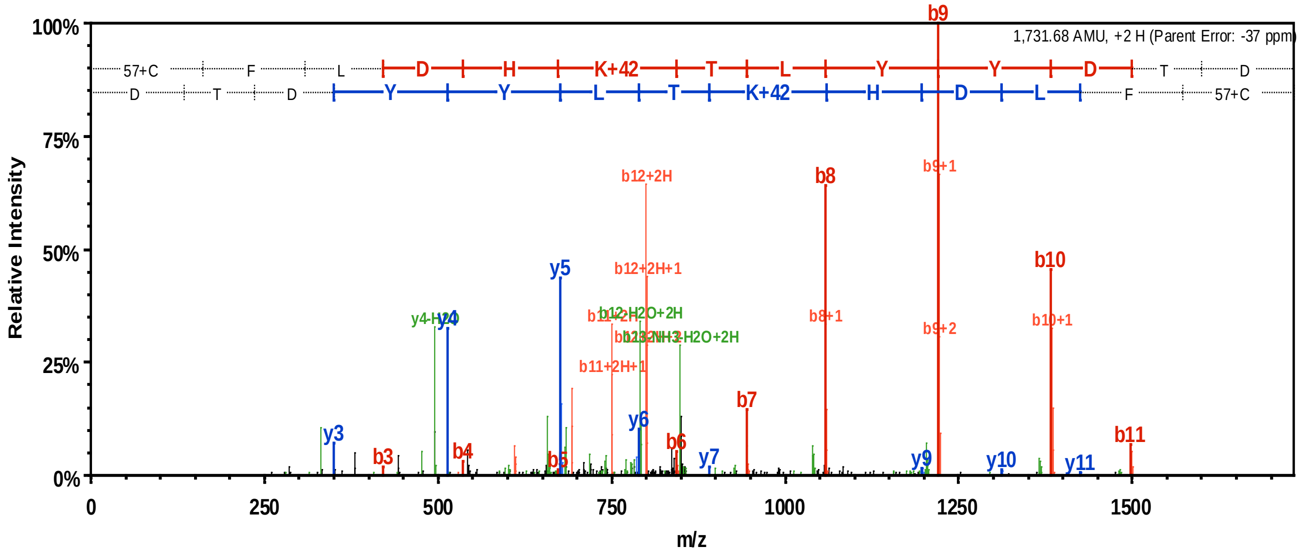

B	B Ions	B+2H	B-NH3	B-H2O	AA	Y Ions	Y+2H	Y-NH3	Y-H2O	Y
1	161.0				C+57	1,732.8	866.9	1,715.7	1,714.7	13
2	308.1				F	1,572.7	786.9	1,555.7	1,554.7	12
3	421.2				L	1,425.7	713.3	1,408.6	1,407.6	11
4	536.2			518.2	D	1,312.6	656.8	1,295.5	1,294.6	10
5	673.3	337.1		655.3	H	1,197.5	599.3	1,180.5	1,179.5	9
6	843.4	422.2	826.4	825.4	K+42	1,060.5	530.7	1,043.5	1,042.5	8
7	944.4	472.7	927.4	926.4	T	890.4			872.4	7
8	1,057.5	529.3	1,040.5	1,039.5	L	789.3			771.3	6
9	1,220.6	610.8	1,203.6	1,202.6	Y	676.2			658.2	5
10	1,383.6	692.3	1,366.6	1,365.6	Y	513.2			495.2	4
11	1,498.7	749.8	1,481.6	1,480.7	D	350.1			332.1	3
12	1,599.7	800.4	1,582.7	1,581.7	T	235.1			217.1	2
13	1,732.8	866.9	1,715.7	1,714.7	D	134.0			116.0	1

Figure S9. LC-MS/MS analysis of the AA(320-334) peptide sequence showing Tip60 is acetylated at lysine 327.

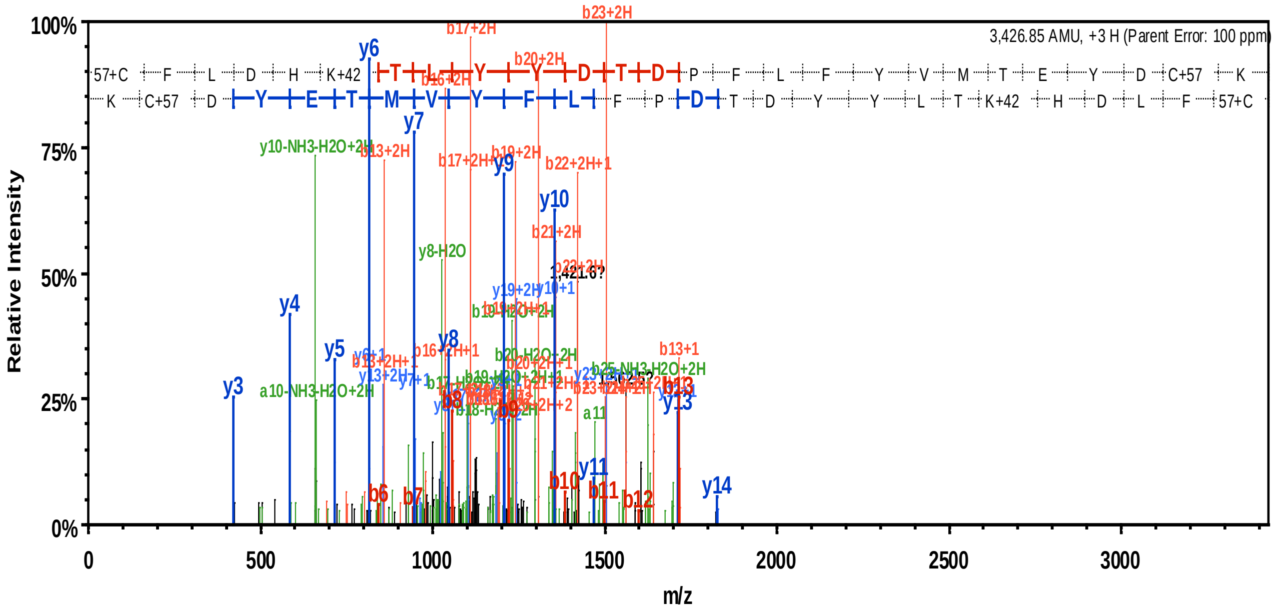

B	B Ions	B+2H	B-NH3	B-H2O	AA	Y Ions	Y+2H	Y-NH3	Y-H2O	Y
1	161.0				C+57	3,426.5	1,713.8	3,409.5	3,408.5	26
2	308.1				F	3,266.5	1,633.7	3,249.4	3,248.5	25
3	421.2				L	3,119.4	1,560.2	3,102.4	3,101.4	24
4	536.2			518.2	D	3,006.3	1,503.7	2,989.3	2,988.3	23
5	673.3	337.1		655.3	H	2,891.3	1,446.2	2,874.3	2,873.3	22
6	843.4	422.2	826.4	825.4	K+42	2,754.2	1,377.6	2,737.2	2,736.2	21
7	944.4	472.7	927.4	926.4	T	2,584.1	1,292.6	2,567.1	2,566.1	20
8	1,057.5	529.3	1,040.5	1,039.5	L	2,483.1	1,242.0	2,466.1	2,465.1	19
9	1,220.6	610.8	1,203.6	1,202.6	Y	2,370.0	1,185.5	2,353.0	2,352.0	18
10	1,383.6	692.3	1,366.6	1,365.6	Y	2,206.9	1,104.0	2,189.9	2,188.9	17
11	1,498.7	749.8	1,481.6	1,480.7	D	2,043.9	1,022.4	2,026.8	2,025.9	16
12	1,599.7	800.4	1,582.7	1,581.7	T	1,928.8	964.9	1,911.8	1,910.8	15
13	1,714.7	857.9	1,697.7	1,696.7	D	1,827.8	914.4	1,810.8	1,809.8	14
14	1,811.8	906.4	1,794.8	1,793.8	P	1,712.8	856.9	1,695.7	1,694.8	13
15	1,958.9	979.9	1,941.8	1,940.9	F	1,615.7	808.4	1,598.7	1,597.7	12
16	2,071.9	1,036.5	2,054.9	2,053.9	L	1,468.6	734.8	1,451.6	1,450.6	11
17	2,219.0	1,110.0	2,202.0	2,201.0	F	1,355.6	678.3	1,338.5	1,337.6	10
18	2,382.1	1,191.5	2,365.1	2,364.1	Y	1,208.5	604.8	1,191.5	1,190.5	9
19	2,481.1	1,241.1	2,464.1	2,463.1	V	1,045.4	523.2	1,028.4	1,027.4	8
20	2,612.2	1,306.6	2,595.2	2,594.2	M	946.4	473.7	929.3	928.4	7
21	2,713.2	1,357.1	2,696.2	2,695.2	T	815.3	408.2	798.3	797.3	6
22	2,842.3	1,421.6	2,825.3	2,824.3	E	714.3		697.2	696.3	5
23	3,005.3	1,503.2	2,988.3	2,987.3	Y	585.2		568.2	567.2	4
24	3,120.4	1,560.7	3,103.3	3,102.4	D	422.2		405.1	404.2	3
25	3,280.4	1,640.7	3,263.4	3,262.4	C+57	307.1		290.1		2
26	3,426.5	1,713.8	3,409.5	3,408.5	K	147.1		130.1		1

Figure S10. LC-MS/MS analysis of the AA(320-340) peptide sequence showing Tip60 is acetylated at lysine 327.

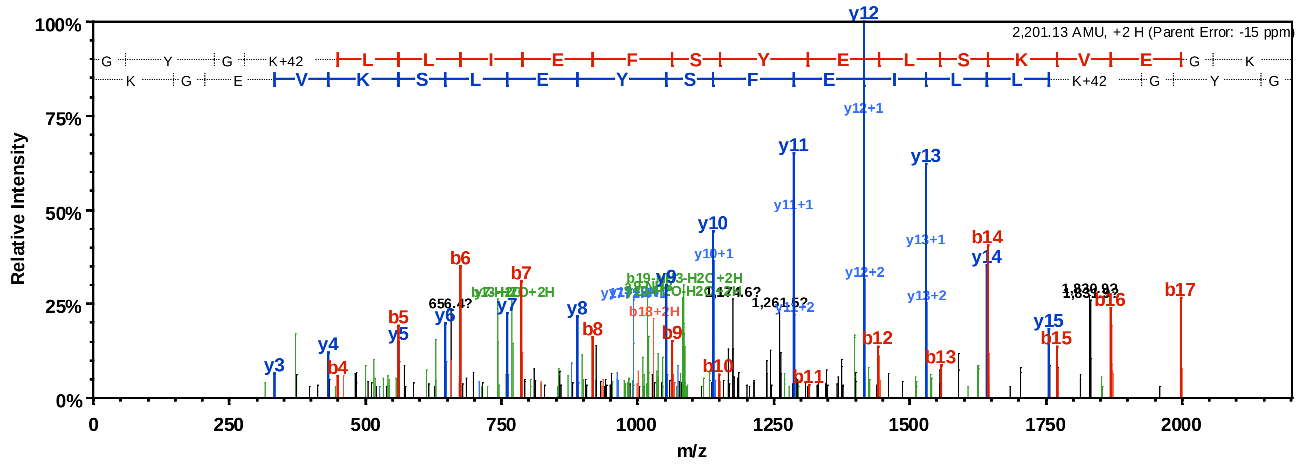

B	B Ions	B+2H	B-NH3	B-H2O	AA	Y Ions	Y+2H	Y-NH3	Y-H2O	Y
1	58.0				G	2,202.2	1,101.6	2,185.1	2,184.2	19
2	221.1				Y	2,145.1	1,073.1	2,128.1	2,127.1	18
3	278.1				G	1,982.1	991.5	1,965.1	1,964.1	17
4	448.2	224.6	431.2		K+42	1,925.1	963.0	1,908.0	1,907.1	16
5	561.3	281.2	544.3		L	1,755.0	878.0	1,737.9	1,736.9	15
6	674.4	337.7	657.4		L	1,641.9	821.4	1,624.8	1,623.9	14
7	787.5	394.2	770.4		I	1,528.8	764.9	1,511.8	1,510.8	13
8	916.5	458.8	899.5	898.5	E	1,415.7	708.4	1,398.7	1,397.7	12
9	1,063.6	532.3	1,046.6	1,045.6	F	1,286.7	643.8	1,269.6	1,268.7	11
10	1,150.6	575.8	1,133.6	1,132.6	S	1,139.6	570.3	1,122.6	1,121.6	10
11	1,313.7	657.3	1,296.7	1,295.7	Y	1,052.6	526.8	1,035.5	1,034.6	9
12	1,442.7	721.9	1,425.7	1,424.7	E	889.5	445.3	872.5	871.5	8
13	1,555.8	778.4	1,538.8	1,537.8	L	760.5	380.7	743.4	742.4	7
14	1,642.8	821.9	1,625.8	1,624.8	S	647.4	324.2	630.3	629.4	6
15	1,770.9	886.0	1,753.9	1,752.9	K	560.3	280.7	543.3	542.3	5
16	1,870.0	935.5	1,853.0	1,852.0	V	432.2		415.2	414.2	4
17	1,999.0	1,000.0	1,982.0	1,981.0	E	333.2		316.2	315.2	3
18	2,056.1	1,028.5	2,039.0	2,038.1	G	204.1		187.1		2
19	2,202.2	1,101.6	2,185.1	2,184.2	K	147.1		130.1		1

Figure S11. LC-MS/MS analysis of the AA(380-398) peptide sequence showing Tip60 is acetylated at lysine 383.
